# Osimertinib induced cardiomyopathy: A case report

**DOI:** 10.1097/MD.0000000000022301

**Published:** 2020-09-25

**Authors:** Shun Shinomiya, Kyoichi Kaira, Ou Yamaguchi, Keitaro Ishikawa, Hiroshi Kagamu

**Affiliations:** Department of Respiratory Medicine, Comprehensive Cancer Center, International Medical Center, Saitama University Hospital, 1397-1 Yamane, Hidaka-City, Saitama, Japan.

**Keywords:** cardiac dysfunction, cardiomyopathy, lung cancer, osimertinib

## Abstract

**Rationale:**

Cardiotoxicity related to osimertinib, including cardiac failure, QT prolongation, and atrial fibrillation, has been reported as an extremely rare incidence in patients with advanced non-small cell lung cancer (NSCLC). However, little is known about the occurrence of osimertinib-induced cardiomyopathy.

**Patient concerns:**

A 76-year old woman was treated with afatinib (40 mg/day) as the 1st line treatment due to recurrence after surgical resection for pulmonary adenocarcinoma. However, she experienced recurrence with positive T790 M, and osimertinib (80 mg/day) was administered as the 2nd line therapy.

**Diagnosis:**

Four months after osimertinib initiation, she complained of fever and progressive dyspnea, and a diagnostic endomyocardial biopsy confirmed non-specific cardiomyopathy, indicating osimertinib-induced cardiomyopathy.

**Interventions and outcomes:**

She was treated with furosemide, carvedilol, and enalapril, and her cardiac function, her symptoms, and condition improved 3 weeks after the withdrawal of osimertinib.

**Lessons:**

Physicians should be alert of the cardiomyopathy-causing potential of osimertinib in advanced NSCLC patients.

## 1 Introduction

Osimertinib is a 3rd generation epidermal growth factor receptor (EGFR) tyrosine kinase inhibitor (TKI), proven effective as 1st line treatment in patients with advanced non-small cell lung cancer (NSCLC) harboring the *EGFR* mutation.^[1]^ As an EGFR-TKI, osimertinib has reported milder toxicities compared with the other EGFR-TKIs such as gefitinib, erlotinib, and afatinib. Recently, some studies have reported cardiac dysfunction as an adverse event secondary to osimertinib.^[2–4]^ The frequency of cardiac dysfunction is less than 1.0%.^[2]^ Hence, it has been classified as an extremely rare incidence. However, physicians should remain alert to the potential of cardiac toxicity resulting from osimertinib treatment. Here, we presented a case of osimertinib-induced cardiomyopathy in a patient with advanced NSCLC.

## 2 Case report

**Figure 1:**
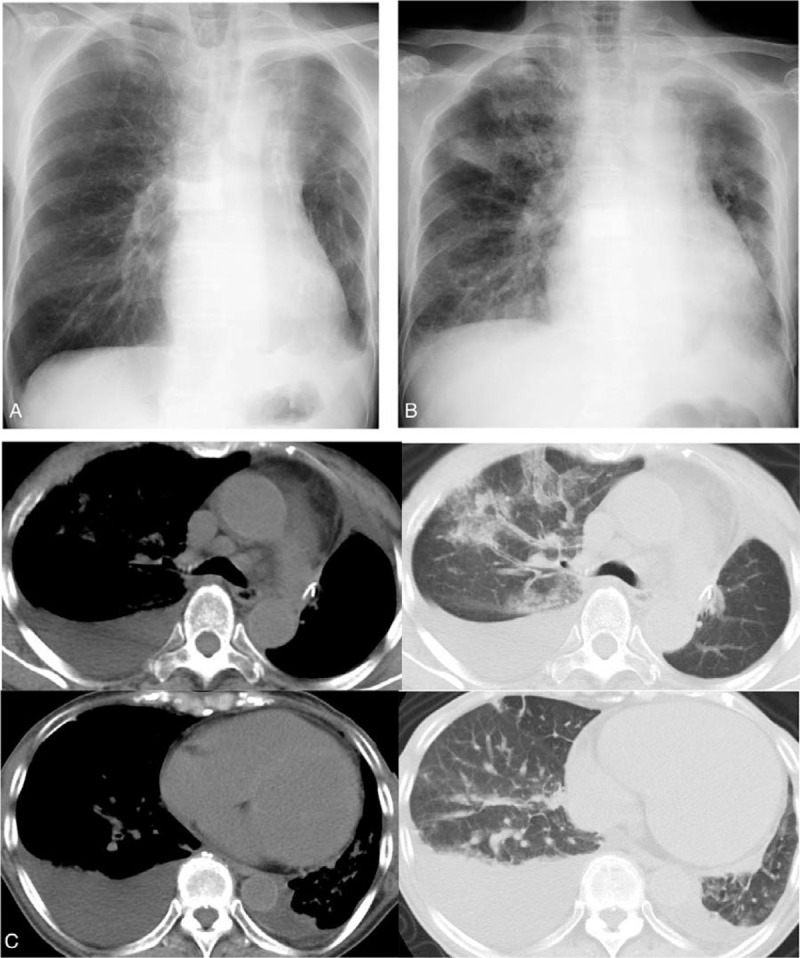
Chest radiography indicates an enlarged cardiac shadow and diffuse ground-glass opacities in both lung fields on admission (A). After osimertinib discontinuation, the chest radiograph displays an improvement in enlarged cardiac shadow (B). Chest CT exhibits cardiac enlargement, pleural effusion, and ground-glass opacities in both lung fields (C).

**Figure 2:**
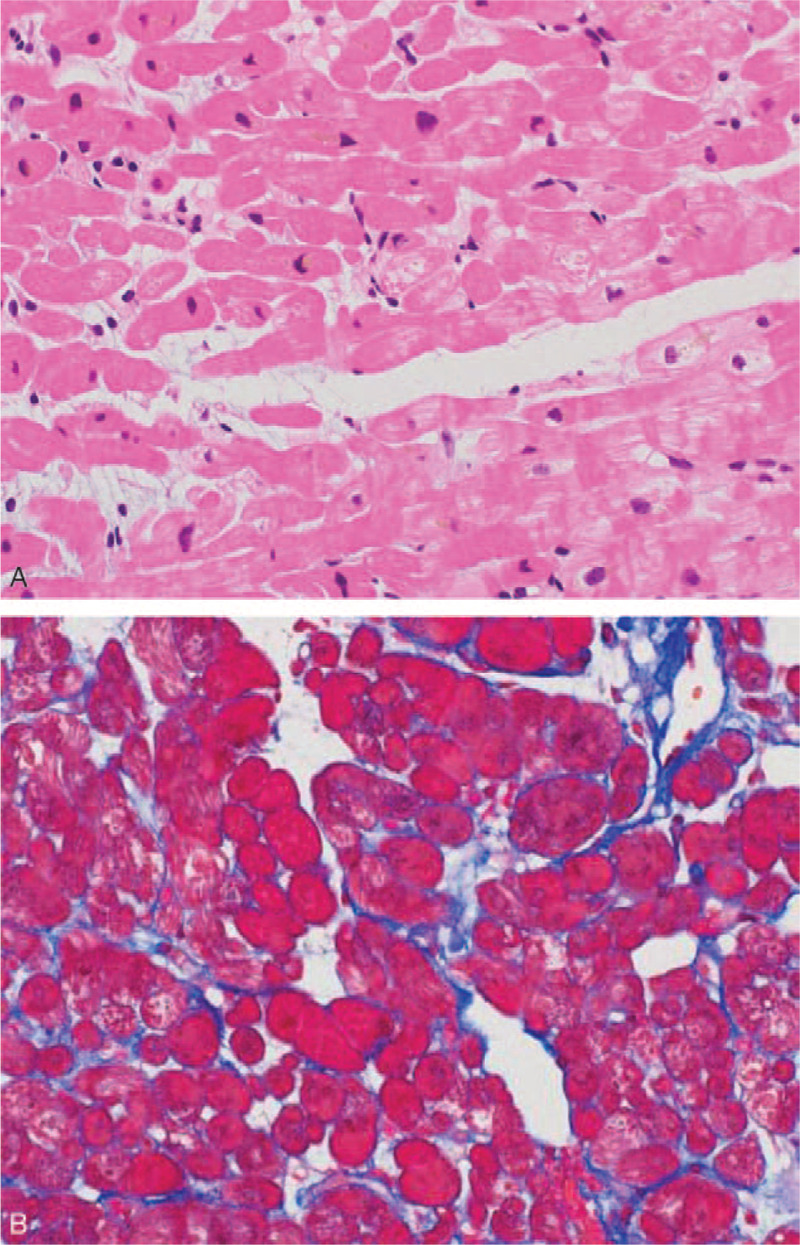
Endomyocardial biopsy of the right ventricle shows non-specific cardiomyopathy, without inflammatory cell infiltration, amyloid deposits, and necrosis. There is evidence of karyotypic irregularities, lipofuscin deposit, cytoplasmic airborne degeneration, and slight interstitial fibrosis. (A) HE and (B) Massons trichrome immunostaining.

A 76-year old female underwent surgical resection of the left upper lobe plus lymph node dissection, 4 years ago following a diagnosis of primary lung adenocarcinoma, harboring an epidermal growth factor receptor (EGFR) deletion mutation in exon 19. One month after surgery, the patient received adjuvant chemotherapy with cisplatin plus vinorelbine. Three years after surgical resection, evidence of bone metastases was noted. Therefore, the patient was initially treated with afatinib 40 mg once daily orally. She experienced recurrence with the T790 M *EGFR* mutation 2 years after afatinib initiation. Hence, osimertinib 80 mg once daily orally was administered. Four months after osimertinib initiation, she complained of fever and progressive dyspnea and was hospitalized at our institution. She had no prior smoking history, and physical examination revealed coarse crackles in both lung fields without any murmur and external edema. Laboratory investigations on admission reported an increased level of brain natriuretic peptide of 1394 pg/ml and troponin I of 40.9 pg/ml. However, no abnormalities concerning antinuclear antibody (ANA), rheumatoid factor (RF), anti-neutrophil cytoplasmic antibody (ANCA), angiotensin-converting enzyme (ACE), IgG4, coxsackievirus, echovirus, and adenovirus were observed. The chest radiography displayed an enlarged cardiac shadow and diffused ground-glass opacities in both lung fields (Fig. 1). The electrocardiogram demonstrated tachycardia without QT prolongation. The chest computed tomography (CT) revealed cardiac enlargement, pleural effusion, and ground-glass opacities in both lung fields (Fig. 1). Furthermore, the echocardiogram indicated an obvious finding of severe hypokinesis, with an ejection fraction of 17% and left ventricular enlargement. Moreover, coronary angiography revealed normal study without ischemic change. A diagnostic endomyocardial biopsy confirmed non-specific cardiomyopathy, without inflammatory cell infiltration, amyloid deposits, and necrosis (Fig. 2). As we strongly suspected the potential of osimertinib-induced cardiomyopathy with CTCAE grade 3, osimertinib was discontinued. The patient was treated with furosemide, carvedilol, and enalapril, and her cardiac function and condition improved 3 weeks after osimertinib withdrawal. The finding of echocardiogram revealed normal study and there was reversal of the cardiomyopathy. Moreover, there were no cardiovascular risk factors that would have predisposed to cardiomyopathy in our patient. Therefore, cardiomyopathy secondary to osimertinib was diagnosed.

## 3 Discussion

Previously, from 2016 to 2018, a rare incidence (3.7%) had been reported regarding the occurrence of cardiotoxicity linked to EGFR-TKIs (osimertinib, erlotinib, afatinib, or gefitinib) based on the data from 8450 adverse events (AEs).^[5]^ Although cardiac failure, atrial fibrillation, QT prolongation, myocardial infarction, and pericardial effusion have already been reported as cardiac toxicities related to EGFR-TKIs, the frequency (6.1%) of cardiac AEs due to osimertinib was high compared to that (2.1%) of other EGFR-TKIs.^[5]^ Notably, osimertinib reported an increased risk of cardiac failure compared to other reported cardiac AEs. However, the underlying mechanism of osimertinib-related cardiotoxicity remains unclear. Notably, it has been reported that 7 cases have experienced cardiac dysfunction related to osimertinib treatment (Table 1).^[2–4]^ Of the 8 cases, including our patient, 7 were female, 6 had an exon 19 *EGFR* sensitive mutation, and osimertinib was administered to 7 cases as 2nd or greater line of treatment. An endomyocardial biopsy was performed in 2 of the 8 cases. Therefore, little information is available regarding pathological findings. Our case indicated non-specific cardiomyopathy, with consistent findings in the echocardiogram, chest radiography, and cardiac examinations. Furthermore, after the discontinuation of osimertinib, our patient improved. Cardiac toxicities have been classified as 2 types, reversible and irreversible. Little is known about the detailed features of cardiac toxicity due to osimertinib. However, our patient demonstrated reversible cardiotoxicity. Although 4 previous cases received retreatment with osimertinib (Table 1), it remains unclear whether retreatment with osimertinib should be undertaken after discontinuation due to cardiotoxicity.

**Table 1:**
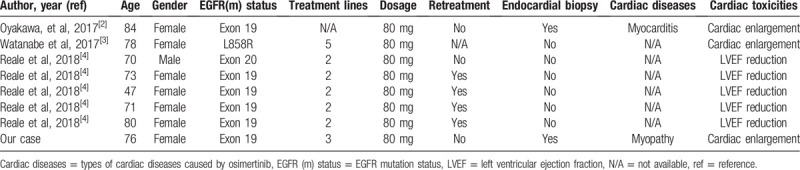
Previous reports regarding cardiac dysfunction due to osimertinib.

Regarding cardiac toxicities associated with osimertinib, a previous investigation reported the incidence of cardiac failure (1.6%), arterial fibrillation (1.2%), QT prolongation (1.3%), and cardiac failure (0.7%).^[5]^ Trastuzumab, a monoclonal antibody against human epidermal growth factor receptor 2 (HER2), has been linked to the occurrence of cardiotoxicity. Although HER2 signaling is necessary for maintaining cardiac function, osimertinib, like trastuzumab, also inhibits the HER2 pathway.^[6]^ Therefore, cardiotoxicity related to osimertinib could occur, albeit rarely. Oncologists should take considerable caution regarding cardiac accidents associated with cancer molecular targeting agents. Recently, we reported a case with ventricular fibrillation followed by QT prolongation attributed to osimertinib administration, with sudden cardiopulmonary arrest, needing cardiopulmonary resuscitation.^[7]^ Hence, an awareness of the life-threatening cardiotoxicity related to osimertinib is crucial. In the present case, evidence of congestive heart failure associated with cardiomyopathy was observed without severe arrhythmia. We hypothesize that cardiomyopathy may be identified as the reason for cardiac dysfunction linked to osimertinib, including reports in previous case series.

In conclusion, physicians should be alert of the cardiomyopathy potential as one of the cardiotoxicities associated with osimertinib administration in patients with advanced NSCLC.

## Acknowledgments

The authors thank Dr. Tsugumi Sato at the Department of Diagnostic Pathology, International Medical Center, Saitama Medical University.

## Author contribution

**Project administration:** Kyoichi Kaira, Shun Shinomiya, Ou Yamaguchi.

**Supervision:** Kyoichi Kaira, Hiroshi Kagamu.

**Validation:** Keitaro Ishikawa, Ou Yamaguchi.

**Writing – original draft:** Kyoichi Kaira, Shun Shinomiya.

**Writing – review & editing:** Hiroshi Kagamu.
